# Constructing a cohesive pattern for collective navigation based on a swarm of robotics

**DOI:** 10.7717/peerj-cs.626

**Published:** 2021-07-27

**Authors:** Yehia A. Soliman, Sarah N. Abdulkader, Taha M. Mohamed

**Affiliations:** 1 Faculty of Computers and Artificial Intelligence, Helwan University, Cairo, Egypt; 2 Faculty of Computer Studies, Arab Open University, Cairo, Egypt; 3 Faculty of Business, University of Jeddah, Kingdom of Saudi Arabia (KSA)

**Keywords:** Swarm robotics, Mobile robots, Aggregation algorithm, Collective navigation

## Abstract

Swarm robotics carries out complex tasks beyond the power of simple individual robots. Limited capabilities of sensing and communication by simple mobile robots have been essential inspirations for aggregation tasks. Aggregation is crucial behavior when performing complex tasks in swarm robotics systems. Many difficulties are facing the aggregation algorithm. These difficulties are as such: this algorithm has to work under the restrictions of no information about positions, no central control, and only local information interaction among robots. This paper proposed a new aggregation algorithm. This algorithm combined with the wave algorithm to achieve collective navigation and the recruitment strategy. In this work, the aggregation algorithm consists of two main phases: the searching phase, and the surrounding phase. The execution time of the proposed algorithm was analyzed. The experimental results showed that the aggregation time in the proposed algorithm was significantly reduced by 41% compared to other algorithms in the literature. Moreover, we analyzed our results using a one-way analysis of variance. Also, our results showed that the increasing swarm size significantly improved the performance of the group.

## Introduction

Swarm robotics is a research field that studies how mobile robots are organized using the local rules (Trianni & Campo, 2015). Most swarm robotics picks their inspiration from nature swarms (Olaronke et al., 2020), like animals, fish and social insects (Hamann, 2018; Berlinger, Gauci & Nagpal, 2021). Swarm robotics carries out complex tasks beyond the power of simple individual robots. Beni (Nedjah & Junior, 2019) states that simple individual robots should have some features such as non-centralized control and low capabilities. Also, swarm robotics has some attractive characteristics such as fault tolerance, scalability, flexibility, economic efficiency, damage resilience and adaptability (Bayındır, 2016).

Swarm robotics has many critical applications such as agricultural forecasting, mining services, target searching, rescue, cooperative transportation, and military operations. Occasionally, some of these tasks may need a large area, dangerous to people, or time consuming. So, they could be easily performed by using swarm robotics (Schranz et al., 2020; Huang et al., 2019).

Small robots often have limited capabilities of sensing and communication. Additionally, small robots do not know the whole picture of the swarm. Therefore, using a robotics swarm can overcome some limitations of a single small robot (Khaldi & Cherif, 2015). For example, using the swarm could help solve complex tasks that a single robot cannot perform. One additional benefit of a robot swarm is that other robots participating in the swarm could perform the task in case of a single robot failure. So, swarm robotics can be a more robust and efficient way to carry out tasks than a single robot when investigating transport or collective coordination.

In swarm robotics, complex tasks are often divided into subtasks (Junior & Nedjah, 2016). These subtasks are aggregation, recruitment, alignment, and collective movement, respectively. All of the subtasks should be performed sequentially. Aggregation is used to bring robots closer together in a swarm and serve as a starting point for extra tasks, such as communication with a limited range. The second subtask represents the recruitment of robots around the leader, and each robot starts to know its neighbors. While the third subtask is to align all robots with the direction of the leader robot, in which they are to adjust their position. Finally, the last subtask calls collective movement, where all robots are moving together from one point to another.

Aggregation (Nedjah & Junior, 2019) is one of the essential bio-inspired behaviors for swarm robots, particularly in collective navigation and transport, and has many real-world applications. Aggregation aims to spatially grouping all robots in a region of the environment (Jiang, 2021). It is considered as a prerequisite, or a first step, for many swarm robotics applications. One of the applications includes collective movement where robots swarm move in a particular pattern (Silva Junior & Nedjah, 2017). Also, collective transport allows robots to transfer heavy objects from one place to another. Unfortunately, aggregation is a complex problem in swarm robotics due to missing a global agent control (Shlyakhov, Vatamaniuk & Ronzhin, 2017). Therefore, it is essential to achieve aggregation by using local information interaction without global control (Hasselmann & Birattari, 2020). Aggregation could be addressed either as a standalone problem or in the context of some other swarm robotics tasks (Bayındır, 2016).

In the Wave algorithm proposed in Silva Junior & Nedjah (2017), the authors assumed that the robots are previously connected in a network. So, no aggregation is required in this case. However, this assumption is not always correct. That is, the Wave algorithm can only work when robots are aggregated in a commonplace. If these robots are distributed in the environment, the Wave algorithm cannot be applied in this case. So, an aggregation is required as previous step to deal with this shortage of the Wave algorithm.

In this paper, we propose a novel aggregation algorithm for swarm robotics. The proposed algorithm could be combined with the Wave algorithm proposed in Silva Junior & Nedjah (2017) to construct a cohesive pattern for collective navigation (Oh et al., 2017). The proposed aggregation algorithm uses the finite state machine (FSM). Moreover, the paper combines the aggregation process with the recruitment process. The recruitment process means that each mobile robot in the swarm can know its neighbors (De Souza e Silva Junior & Nedjah, 2016). The previous achievement avoids the recruitment task, knowing that, the recruitment task is required for the Wave algorithm.

The proposed algorithm works under absence of position information, central control, and global interaction among robots. Additionally, the proposed algorithm performs the recruitment task (De Souza e Silva Junior & Nedjah, 2016). The rest of this paper is organized as follows: “Literature Review” surveys the literature review. “The Proposed Aggregation Algorithm” presents the proposed aggregation algorithm and also the integration with the Wave algorithm. “The Experimental Results” presents the simulation results. Finally, the conclusions are presented in “Conclusions”.

## Literature review

Swarm robotics research includes various tasks such as aggregation, flocking and navigation (Nedjah & Junior, 2019; Bayındır, 2016). There are two distinct mechanisms used by insects to perform aggregation: cue-based aggregation and self-organized aggregation (Arvin et al., 2016; Amjadi, Raoufi & Turgut, 2021). In cue-based aggregation, insects like honeybees are observed to huddle based on the temperature degree at the point of their aggregation. In self-organized aggregation, insects like cockroaches are aggregated without a previous choice of the environmental conditions at their aggregation location. Shlyakhov, Vatamaniuk & Ronzhin (2017) surveys various methods of aggregation. These methods include virtual force, probabilistic algorithms, and artificial evolution. The method of virtual forces is used to model the behavior of robots based on the attractive and repulsive forces’ calculation. In the probabilistic method, every robot has a random component and is adapted in the robot’s interaction approach with the environment (Khaldi et al., 2019). Besides, probabilistic methods are inspired by nature, like cockroaches and bees (Firat et al., 2020; Valentini, Ferrante & Dorigo, 2017). The artificial evolution method is based on the concept of adaptation. Bayındır (2016) demonstrates that the aggregation is constructed using robots based on artificial evolution.

Ramroop et al. (2018) proposed an aggregation algorithm using swarm robotics based on the biological systems. They used the BEECLUST aggregation algorithm, a bio-inspired swarm, where honeybees tend to group in areas with temperatures between 34 and 38 °C. BEECLUST uses sound signals or light intensity instead of temperature to group robots. The more robots used, the less aggregation time is. The aggregation time ranges from 0 to 500 s based on the total number of cues and the number of robots. The aggregation time is decreased using more light sources. Fortunately, our proposed algorithm outperforms the BEECLUST algorithm as shown in the experimental results section.

Arvin et al. (2018) presented an aggregation method based on pheromone. Pheromone has an essential role in swarm robotics communication used for commonplace aggregation. In the first phase, the experiment is performed with the BEECLUST algorithm. The aggregation performance increases as swarm size increases (Arvin et al., 2014b). In the second phase, the pheromone guidance is carried out. The aggregation performance improved with pheromone guidance compared to BEECLUST. The aggregation is guaranteed, but the aggregation time differs depending on the population size. However, robots depend on light intensity. The sensors performance is negatively affected in several ways. The absence of a colony leader complicates a colony trajectory control. The aggregation time ranges from 0 to 450 s based on the robot’s size and pheromone sensing radius. The results show an improvement in the aggregation performance in all configurations when using pheromone (Tang et al., 2017).

Beltran et al. (2018) proposed a collective behavior on ten Kilobots. They presented a target-surrounding method for collective behavior. The target-surrounding method’s success rate is only 60%, due to the Kilobot’s hardware problems while moving. The algorithm uses real robots with different swarm sizes in the experiments. Furthermore, the area should not be directly under a light source. On the contrary, increasing the number of robots increases the time of the aggregation.

Hu et al. (2014) proposed a self-organized aggregation model based on cockroaches’ aggregation behavior. The aggregation depends on the likelihood to join or to leave the grouping. The aggregation grows through local information among individuals without the existence of a central control or a leader. The local message uses the probabilistic finite-state automaton (PFSA). The robots are arranged in aggregation as a multi-tree, radial pattern, or regular polygon. The average time of aggregation equals 911 s when six robots are aggregated in a multi-tree.

Sun et al. (2019) proposed a bio-inspired virtual pheromones system. It was used to mimics the pheromone-based aggregation behavior of insects. The leader robot issues one of the pheromones to sign his followers to aggregate at its current location. Virtual pheromones were simulated using different colors. The experiments were carried out with three robots; a leader robot, predator robot, and follower robots. Whenever a leading robot sent out the pheromone aggregation, the robots moved toward its direction to aggregate. The system has some limitations; the pheromone release takes a little time and the visual tracking algorithm is a little sensitive to the ambient light.

Li et al. (2015) presented the evolutionary algorithm framework for swarm self-assembly using Sambots robots. The framework can produce various shapes. Each robot can walk, communicate, and aggregate independently with other robots. The aggregation time was about 120 s for nine robots to create a four-legged structure.

Dimidov, Oriol & Trianni (2016) used a random walk for swarm movement. The robots used have simple localization and mapping capabilities. The random walk is considered the first step in constructing a collective pattern. They analyzed the efficiency of the random walk algorithm in the Kilobots swarm. The authors use both real and simulated robots with different swarm sizes. Robots can interact with other robots upon encounters. They analyzed the efficiency of random walking patterns for a swarm of Kilobots in two different environments. However, there is no strategy for collision avoidance in their algorithm. That is, robots may block for long time if collides with walls or corners. The algorithm efficiency performance is 75% when using 10–30 robots.

Silva Junior & Nedjah (2017) presented the Wave algorithm. The Wave algorithm forms collective navigation, and this is an essential task in robotic swarm applications. The algorithm is based on local information interactions. The communication is performed by message passing through robot neighbors. Therefore, it is performed through three subtasks, recruitment, alignment, and movement. They assumed that robots are previously grouped in a connected network. Finally, robots can move together in different scenarios with obstacles. The swarm leader facilitates a swarm trajectory control. The collective movement is guaranteed. However, the usage of a leader entity degrades the robustness of the swarm robotic system. Moreover, the Wave algorithm cannot be applied if the robots are not previously organized in a connected topology. Also, the alignment process consumes a lot of time. The time required for movement is not stated. Also, the authors assume a pre-robot grouping (Junior & Nedjah, 2016; Silva Junior & Nedjah, 2017; De Souza e Silva Junior & Nedjah, 2016). However, this assumption is not always correct as we will show later. Interestingly, our proposed algorithm enhances the performance of the Wave algorithm by adding a new aggregation phase to be combined with the recruitment phase.

Regarding simulation platforms, there are several robotic platforms used for swarm robotics simulation. Some of them are Kilobots (Rubenstein et al., 2014), Colias (Arvin et al., 2014a), Spiderino (Jdeed et al., 2017) and Mona (Arvin et al., 2019). Kilobot is one of the low-cost mobile robot. In this paper, the proposed algorithm uses the Kilobot robots and the V-REP simulator (Rohmer, Singh & Freese, 2013) due to their important advantages, and also for comparing our proposed work with the previous work (Ramroop et al., 2018).

From the previous discussion, it is clear that the aggregation process has been studied as a stand-alone problem and not as part of more specialized tasks that involve aggregating several agents. Most of the previous algorithms lack to apply them in real-life applications. Besides, all algorithms nearly take much time for the aggregation task. Also, there are several robotic platforms with different capabilities are used for aggregation.

## The proposed aggregation algorithm

Aggregation is a collective behavior that aims to group some robots in a commonplace. Aggregation is a significant process in swarm robotics systems. It is inspired from social insects’ behavior (Ward & Webster, 2016). Aggregation is essential, especially when using small robots. It is considered as a prerequisite step for more complex collective behaviors. Cue-based aggregation focuses on aggregating robots on a specific place. The proposed algorithm is a cue-based algorithm. In the proposed method, limited sensors robots are aggregated by cooperation. The aim of this paper is to represent a novel aggregation algorithm for swarm robotics. The proposed aggregation uses the Finite State Machine (FSM) (Hamann, 2018).

The finite-state machine (FSM) is a model used to represent and control execution flow (Ash, 2021). Also, it is a model of computation based on a hypothetical machine including one or more states. Only one state is active at a time in FSM. To perform different actions, the machine passes from one state to another. FSM perfectly models swarm robotics. The proposed algorithm satisfies the aggregation through three states: searcher, parent, and child. The algorithm includes two phases: the first phase is the searching phase which includes the searcher state, while the second phase is the surrounding phase which includes both the child and the parent states. FSM could is applied through two phases: searching and surrounding. Each robot transit from one state to another based on local messages. Table 1 show all abbreviations used in the proposed algorithm. The proposed algorithm is illustrated in Algorithm 1.

**Table 1:** Abbreviations used in the proposed algorithm.

Abbreviation	Description
**FSM**	Finite state machine
**msg**	Local Message
**id**	Unique id
**x**	Robot in search state
**y**	Robot in the child state
**p**	Robot in a parent state
**Request-child**	Robot p broadcast message to recruit child robot
**Acknowledge**	Robot x become a child robot to robot p
**Became-parent**	The state of robot y will be changed to the parent state
**R**	Search state
**C**	Child state
**LR**	Leader robot
**d**	the distance among two robots
**r**	the perception range of the robot

**Algorithm 1:** Proposed aggregation algorithm.

**Require**: number of robots neighbors;
code1 = request-child;
code2 = acknowledge;
code3 = became-parent;
1. leader election process();
2. **If** leader **then**
3. send message( robot id, code1);
4. receive feedback message from child robots();
5. **If** No. of child }{}= two **then**
6. send message(robot id, code3);
7. **End if**
8. **else**
9. search state();
10. receive message();
11. **If** msg1 \ne robot id and msg2 = code1 **then**
12. child state and stop moving();
13. send message(robot id, code2);
14. ** else if** msg1 \ne robot id and msg2 = code3 **then**
15. parent state();
16. ** End if**
17. ** End if**

**Figure 1:**
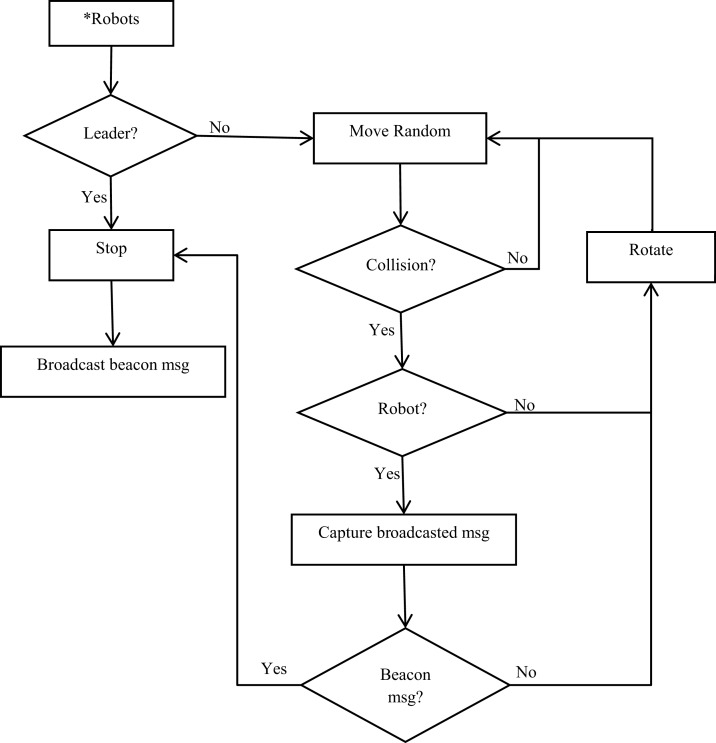
Flowchart of the proposed aggregation algorithm.

Figure 1 shows the flowchart of the proposed aggregation algorithm. Asterisk illustrates the starting state for each robot. Diamonds illustrate control structures (if-else decisions). The robot is in one of six states as follows:

1. Robot: leader election process based on robot (id).
2. Move Random: other robots move randomly while checking for obstacles.
3. Stop: robot stops if it receives the defined beacon message.
4. Obstacle: robot checks if an obstacle is a wall or a robot.
5. Rotate: robot rotates to avoid an obstacle or a robot and then returns to move randomly.
6. Broadcast: robot broadcasts continuous and frequent beacon message.

### Modeling with the FSM

**Figure 2:**
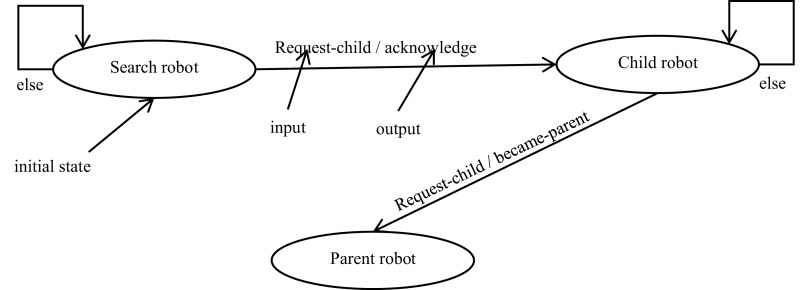
State transition diagram for robot controller.

According to FSM, aggregation is achieved by using different states. The transition from one state to another is based on a local message value. Figure 2 shows the proposed aggregation process. The FSM is initially in its starting search state. When no input is received, the robot stays in the same search state. When a moving robot x receives a request-child message from parent robot p, then the robot x send an acknowledge message to the parent robot p. So, the robot x moves to the next state (child). When the parent has two children, and then sends a become-parent message to its children y. So, these children robots will be allowed to send a request-child message.

Additionally, Table 2 illustrates the inputs and outputs of the different robot states (Ash, 2021). For example, suppose a robot is in the search state and receives an input message, equal *request-child*. In this case, it will transit to the next state called child state and it will send output message equal *acknowledge* according to Eq. (6).

**Table 2:** Robot state transition based on local messages.

Current state	Input	Output	Next state
search	else		search
search	request-child	acknowledge	child
child	became-parent	request-child	parent
child	else		child

The mathematical formulation of the proposed state machine is as follows: e represents the state machine, *M*, is a five-tuple Eq. (1), where states *S*, inputs *I*, and outputs *O* are sets. The update *U* is a function. The initial state *L*\; \; \in *S*. So, M could be represented from Eq. (1) as:

(1) }{}M = \left( {S,I,\; O,\; U,\; L} \right)

The state machine of Fig. 1 has three states. The set of states is as follows:

(2) }{}S = \; \left\{ {R,{\rm \; }C,{\rm \; }P} \right\}

The input and output alphabets of Fig. 1, the sets of input and output are as follows:

(3) }{}I = \; \left\{ {request\; child,\; became\; parent,\; else} \right\}

(4) }{}O = \; \left\{ {acknowledge,\; request\; child,\; else} \right\}

The update function *U* could be represented as follows:

(5) }{}{\rm U}:{\rm \; \; \; \; States\; } \times {\rm Inputs\; } \to {\rm States\; } \times {\rm Outputs}

The initial state is fixed to *l*, which represents the searcher state.

If we consider the current step *k*, then the update function is as follows: If }{}{{\rm s}_{\rm k}} \in S represents the current state at step k,}{}{\rm \; }{{\rm x}_{\rm k}}{\rm \; } \in I is the current input symbol at step *k*. Then, the current output symbol and the next state are given by Eq. (6) as:

(6) \forall {\rm k\; } \ge 0,{\rm \; \; \; \; \; \; \; \; \; \; \; \; \; \; \; \; }\left( {{{\rm s}_{{\rm k} + 1}},{{\rm y}_{\rm k}}} \right){\rm \; } = {\rm U}\left( {{{\rm s}_{\rm k}},{\rm \; }{{\rm x}_{\rm k}}} \right)

Such that,

(7) \forall {\rm k\; } \in {\rm N},{\rm \; \; \; \; \; y}\left( {\rm k} \right) = {\rm \; }\left\{ {\matrix{ {{\rm acknowledge}\; \; \; \; \; {\rm if}\; {x_k} = {\rm request\; child}} \cr {\rm request\; child\; \; \; \; \; if}{\; {x_k} = {\rm became\; parent}} \cr {\rm else}{\; \; \; \; \; \; \; \; \; \; \; \; \; \; \; \; \; \; \; \; \; \; \; \; \; \; {\rm otherwise}} \cr } } \right.{\rm \; \; \; \; \; \; \; \; \; \; \; \; \; \; \; }

Where *N* is the set of natural numbers. FSM could be subdivided into two main phases: searching and surrounding. These two phases are detailed next.

#### The searching phase

The searching phase represents the search state of the finite state machine. The searching phase is crucial for robots to be grouped about their leader. In swarm robotics, every robot has a unique ID (*Robot ID*) (Hu et al., 2014). Due to the limited capability of Kilobot robots, we use the random walk algorithm in this phase due to its simplicity (Pang et al., 2019). Besides, the random walk is used to characterize insects’ movement distinguished by a sequence of straight motion and turns (Wang et al., 2020). All robots move randomly at a constant speed and continuously investigate the environment using their sensory system. In the random walk algorithm, all robots are initially distributed in the area. A robot moves randomly until it receives a message (request-child) from the target object (parent). Then, it will stop immediately and send messages (acknowledge). In case the robot detects an obstacle, it changes its direction to avoid the collision.

**Figure 3:**
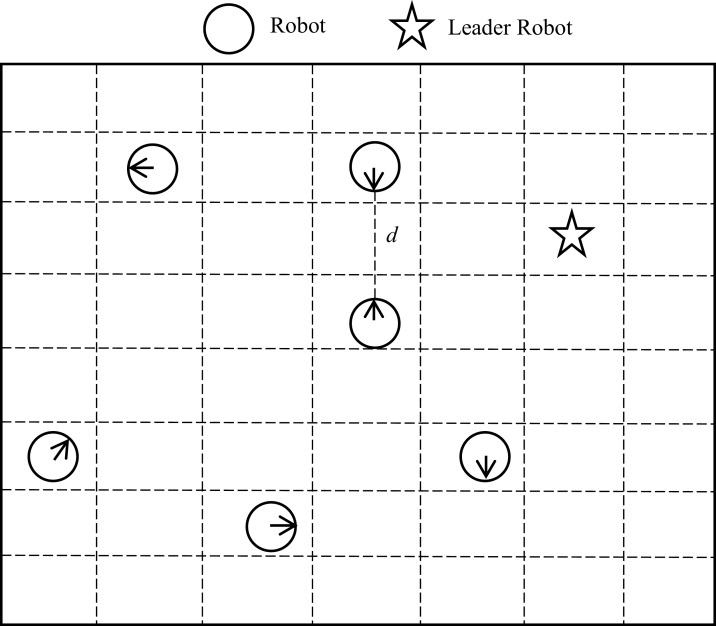
Initial workspace of the searching phase.

Figure 3 is a simulation of the initial workspace. The robots are distributed randomly in the area. In Fig. 3 *star* represent a leader robot. The distance between two robots that can be communicating and sensing is represented by ‘*d*’. Robots use sensors to determine their neighbors. During this phase, all robots move randomly in the area except the leader robot. That is, the leader robot only sends and receives messages. When the leader robot has two children, it will be idle in this case. So, each robot of the two children changes their state to parents to send and receive messages. The process is repeated for all robots in the swarm.

#### The surrounding phase

This phase aims to organize all robots in a form of logical binary tree. The distance between any two adjacent robots is no more than the sensing range of each robot in the arrangement. A robot decides to join the aggregation if the message is equal to the message (request-child). Next, the leader will be idle. Then, each of the two children robots will transit to the parent state. When repeating this process, the final obtained arrangement is a binary tree form.

**Figure 4:**
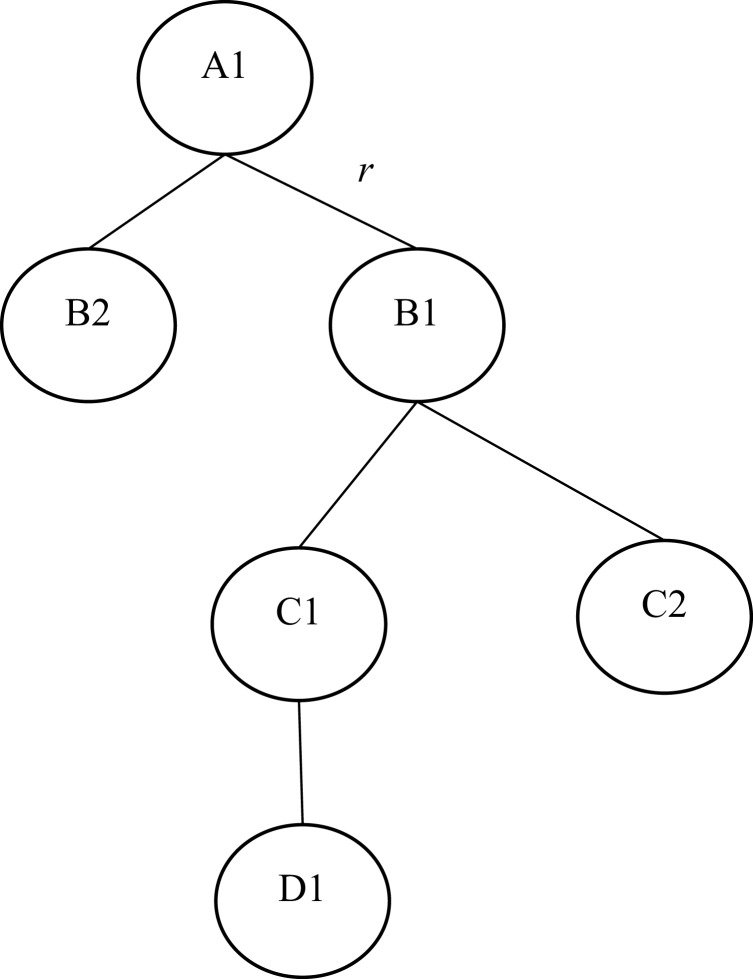
Arrangement of robots in a binary tree form.

Figure 4 illustrates an example of the surrounding phase. In Fig. 4, A is the leader robot, and B1 and B2 are the child robots of A. While, C1 and C2 are the child robots of B1 and D1 is the child robot of C1. B1 and C1 are parent robots, while B2, C2, and D1 are leaf robots. r is the perception range of the robot. The first robot of the tree is the leader. If another robot connects to this leader, the leader becomes the parent. The connected robot becomes the child robot in this case.

### Combined the aggregation algorithm with the wave algorithm

The Wave algorithm proposed in Silva Junior & Nedjah (2017) is a distributed algorithm based on a network of connected robots. The Wave algorithm consists of three main phases: recruitment, alignment, and collective movement. So, we aim to combine our proposed aggregation algorithm with the Wave algorithm. In the Wave algorithm, the authors assumed that the robots are previously connected in a network, and no aggregation is required (Silva Junior & Nedjah, 2017). This assumption is not always correct because the Wave algorithm works on aggregated robots in commonplace. On the contrary, if robots are distributed in the environment, the Wave algorithm cannot be applied in this case. The Wave algorithm uses message passing for swarm communication. The Wave algorithm uses the Kilobot robots. Each Kilobot can communicate at a rate up to 30 kb/s. The distance between any two robots is up to 10 cm (Rubenstein et al., 2014).

**Figure 5:**
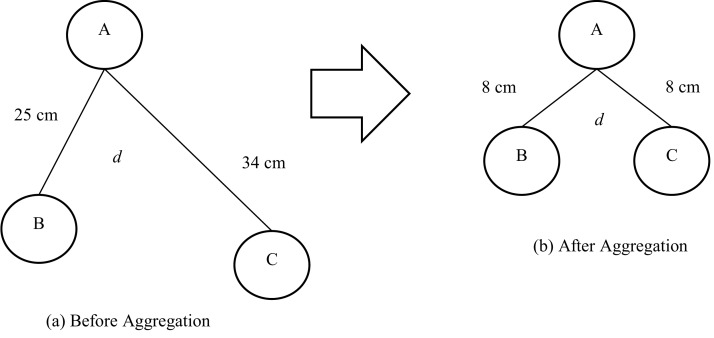
Network of robots before and after aggregation.

To illustrate this limitation of the Wave algorithm, consider three robots A, B and C shown in Fig. 5. These robots need to move together in a coordinated manner. The initial positions of the robots are illustrated in Fig. 5A. The distance between robot A and robot B is 25 cm. The distance between robot A and robot C is 34 cm. Robot A is the leader in this case. So, if the Wave algorithm is applied directly, it will fail to form a swarm due to the long distance between robot A and the two robots B and C.

However, applying our proposed algorithm on the Fig. 5A will give the shape shown in Fig. 5B. So, the aggregation is completed making robot (A) a leader. Additionally, our proposed algorithm gives the surrounding phase a replacement of the Wave algorithms recruitment phase. The two remaining phases of the Wave algorithm (alignment and movement) could be applied to form the collective movement. So, the proposed algorithm may be considered as a necessary preprocessing step of the Wave algorithm to be valid in all cases. Also, the proposed algorithm replaces the recruitment phase of the Wave algorithm by the proposed surrounding phase.

## The experimental results

**Figure 6:**
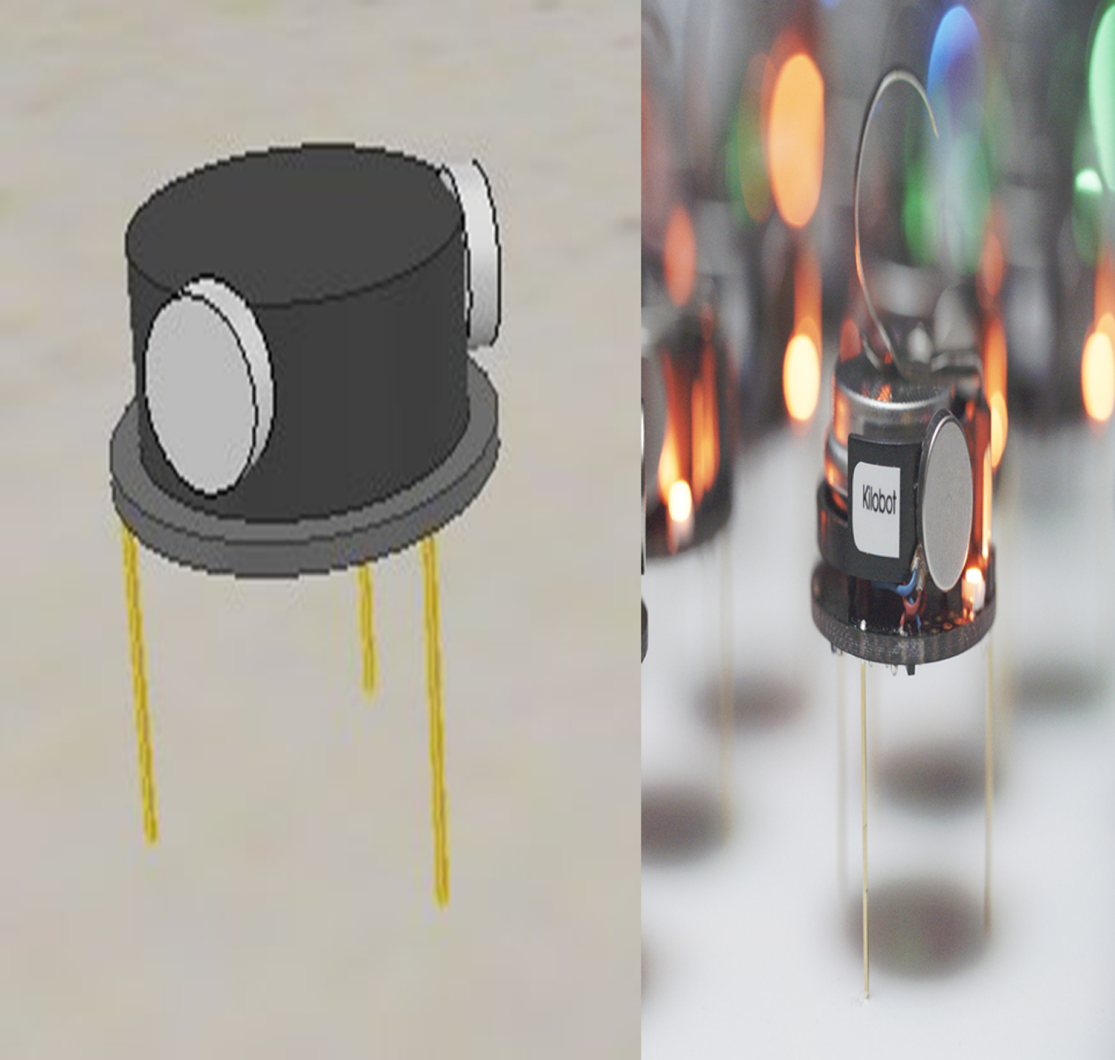
Both simulated and real Kilobot.

Kilobot (Rubenstein et al., 2014) is a robotic platform that has been developed for use in swarm robotics. Kilobots are used as testbed because of their attractive operational scalability and low price. Figure 6 shows both a real and simulated Kilobot. Each Kilobot can move around and keep away from obstacles in the area using a proximity sensor. Also, it has a neighbor-to-neighbor communication and an ambient light sensing.

Our experiments were performed using the V-REP robotic platform (Freese, 2021) with simulation parameters illustrated in Table 3. V-REP is an open-source platform and has 3D models that create, compose and simulates many robotic systems (Zahugi, Prasad & Prasad, 2012). We used the V-REP simulator for its supports of all features of the real Kilobot robots. All experiments were performed on a PC with Windows (7) and an Intel Core i3 processor with 4 GB RAM. We evaluated the aggregation computation time in comparison with the BEECLUST developed by Ramroop et al. (2018). We also calculated the computation of the number of robots aggregated over time for *n* = 4 and *n* = 8.

**Table 3:** Simulation parameters.

Parameters	Range/value
Swarm size of robots	{3,6,9,12,15}
Number of aggregated robots	0–15
Area size	50 × 50 cm
Forward speed	1 cm/s
Turn speed	45 deg/sec
Radius communication	10 cm
Sensor value	0–255
Aggregation time	0–400 s

### Aggregation experiments

In this experiment, we test the required time to complete the aggregation task. Our experiments were conducted using five different scenarios. Each scenario was simulated five times for greater accuracy and the average value of the aggregation time was calculated from each scenario as illustrated in Table 4. All robots started from random positions and orientations. The aggregation time was recorded. A robot was considered to be aggregated if it was received a message with request-child. The aggregation is considered completed when 90% of robots are aggregated. We also compared the aggregation time of the proposed algorithm with BEECLUST algorithm (Ramroop et al., 2018).

**Table 4:** Aggregation time of each scenario in seconds.

Scenario	Avg. “Proposed Algorithm”	Avg. “BEECLUST Algorithm (Ramroop et al., 2018)”
3R	371	583
6R	282	466
9R	240	376
12R	175	332
15R	102	242

#### Results of aggregation time

**Figure 7:**
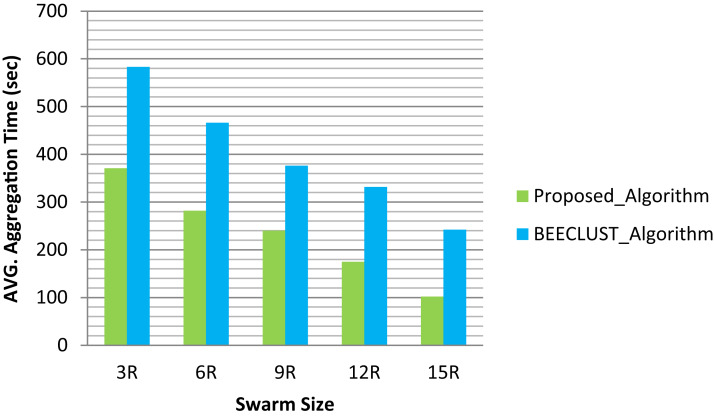
Aggregation time with different swarm sizes.

In Fig. 7 presented for analysis, we can observe the aggregation time of the proposed algorithm compared to the BEECLUST algorithm for the different number of robots in swarm size. We can clearly see that the aggregation time of the proposed algorithm less than the aggregation time of the BEECLUST algorithm in all swarm sizes. The reason is that our aggregation algorithm depends on local information interaction for aggregation to take place. Additionally, the aggregation time of both algorithms decreases by increase the number of robots. As a first specific detail, the results show that the first scenario has the highest average aggregation time, whereas, the fifth scenario has the lowest average aggregation time. Moving on to the different swarm sizes we can see that the aggregation time of the proposed algorithm and BEECLUST algorithm continue to low. Interestingly, this observation matches the observations stated by Ramroop et al. (2018) and Arvin et al. (2018). Finally, the average aggregation time of the proposed algorithm was enhanced by 41% compared to the BEECLUST algorithm. Our proposed algorithm was the highest performing in the five scenarios studied.

**Figure 8:**
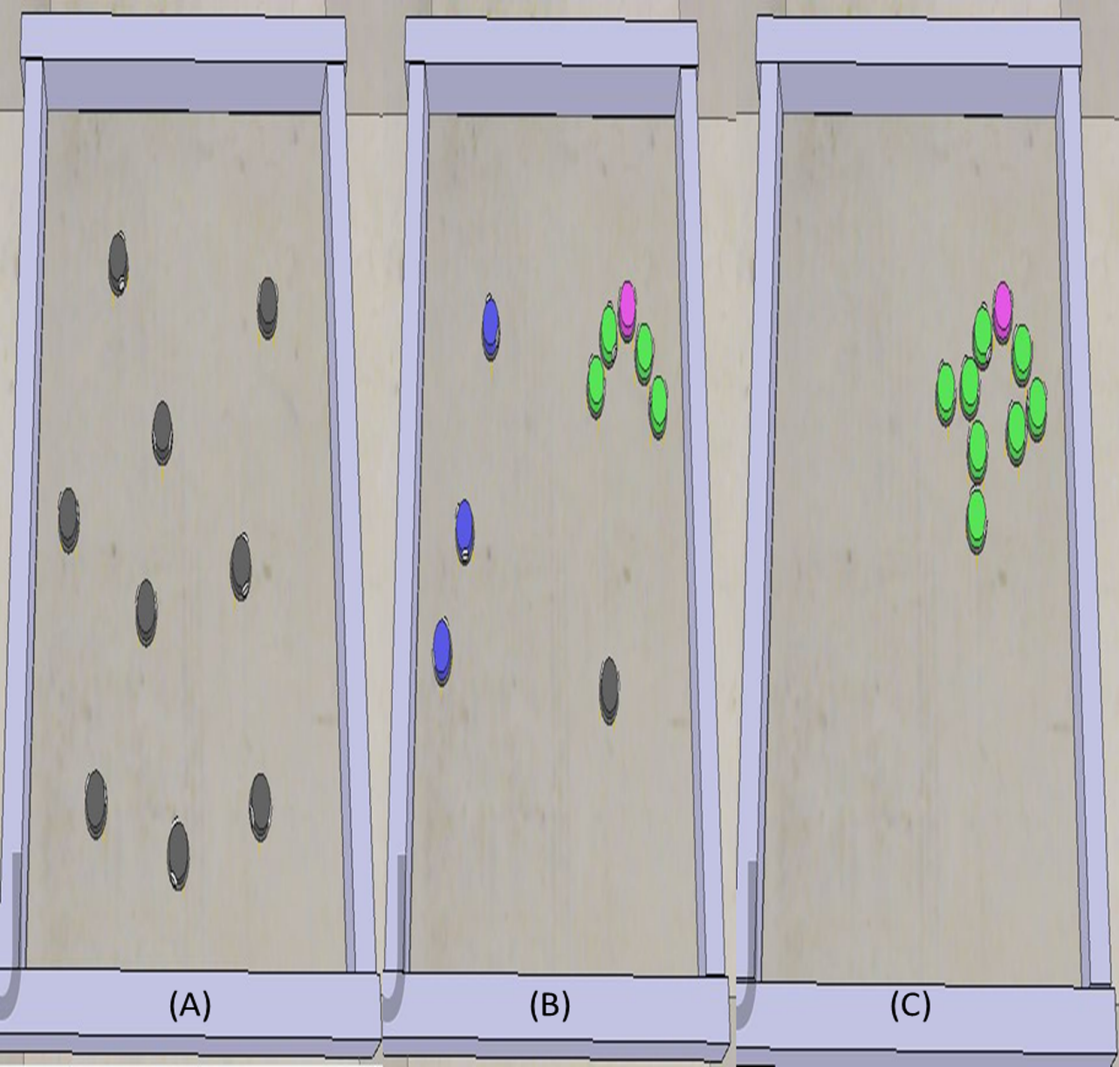
Start of the algorithm from left side and target has been surrounding at the right side.

Figure 8 shows one example scenario. Figure 8A indicates the initial positions of the robots in the environment. Here, the objective of robots is to surround the leader (colored turquoise). Then, the random walk algorithm is executed for each robot. Figure 8B shows the process of aggregating two robots. Here, the green robots will receive a local message and make decisions. Figure 8C shows the result of the proposed aggregation algorithm. So, all robots are aggregated.

#### The impact of the number of robots on the aggregation time

**Figure 9:**
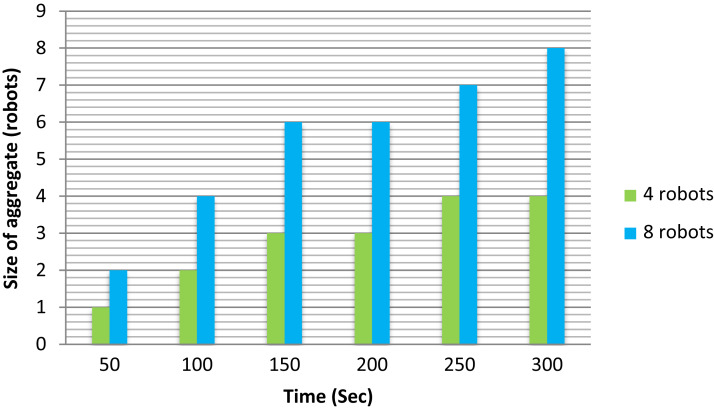
Number of robots in the aggregate over time.

To demonstrate sufficiently the effects of increasing swarm size on the aggregation task. We performed our experiment using two different scenarios. In the first scenario, we used four robots in swarm size, and the second scenario; we used eight robots in swarm size. The experiments were repeated five times for accuracy. As we can see, Fig. 9 illustrates the number of robots aggregated over time for *n* = 4 and *n* = 8, respectively, measured in the robots. In general, we can observe that at time 50 s the aggregate robots in swarm size (8) greater than the swarm size (4). In addition to this clear point, we can also witness that at any different time the aggregated robots in swarm size (8) remain the greatest. Beginning with 50–300 s we can see how the aggregate robots rose over time, but they were rapid in swarm size (8) than swarm size (4). For the case where *n* = 4, the number of robots increases slowly because there is a lower collision rate with aggregated robots. In comparison, for the case where *n* = 8, there is a general increase in the aggregate size over time. Finally, this experiment concluded that a larger aggregate is more likely to increase in size since unaggregated robots have a higher probability of colliding with aggregated robots.

#### Results of statistical analysis

Additionally, the proposed algorithm results are analyzed using a one-way analysis of variance (ANOVA). The F-test method was used to disprove the null hypotheses. The F-value indicates the impact of parameters on the results (factors). There are two key parameters as follows: the F-value and *p*-value of each setting. The F-value indicates the impact of parameters on the results (factors). Factors of higher F value (>1) have more significance. The *p*-value shows how factors influence the results. A low *p*-value (<0.05) indicates that the results are substantially affected by the factors. Otherwise, the factors will not influence the results significantly. The number of robots is the factor and the aggregation time is the response. Table 5 shows the ANOVA results for the proposed algorithm. The results show that the number of robots had a significant (*p* < 0.05) impact on the aggregation performance. Data indicated that in the case of increasing the number of robots, the efficiency increased.

**Table 5:** Statistical analytical results (ANOVA).

Factor	F-value	*p*-value
Number of robots	9.91	0.0001

## Conclusions

Aggregation is a very important process in the field of swarm robotics because it is the first step for implementing a complex task. We proposed a novel aggregation algorithm that uses the concept of the finite state machine. The proposed algorithm has two subtasks; searching and surrounding. The proposed algorithm efficiently reduced the time required for the aggregation process. The proposed aggregation algorithm achieves the recruitment task of the Wave algorithm. Also, the proposed algorithm can solve some cases in which the Wave algorithm cannot be applied. The proposed aggregation algorithm only relies on the local information interaction without global or external control. The algorithm is very suitable for robots with small capabilities like Kilobots. We combined the aggregation algorithm with the Wave algorithm’s alignment and movement; to construct a cohesive pattern for collective navigation. We simulate different simulations with a different number of robots. We analyzed the time consumed on the aggregation task. Results obtained from the simulations show that the aggregation time was significantly reduced using the proposed algorithm. The average aggregation time reduced by 41% in the proposed algorithm compared to other work in the literature. An important observation from the experimental results is that increasing swarm size increases the aggregation performance. When comparing the proposed results with other related work, the proposed aggregation time is always less than other previous work. The results are supported by ANOVA results. The results show some cases where the Wave algorithm cannot be applied, and these cases are solved by the proposed algorithm. The results indicate that the aggregation time decreases as the number of robots in the swarm increases.

## Supplemental Information

10.7717/peerj-cs.626/supp-1 Supplemental Information 1 Source code of the proposed algorithm. To run the aggregation experiment:
1. Download CoppeliaSim V4.2.0 rev5: https://www.coppeliarobotics.com/downloads
2. Go to Tools menu, select File -> open scene -> choose file (Source_Code_of_Proposed_Algorithm)
3. From Simulation -> press start simulation

## Additional Information and Declarations

### Data Availability

The following information was supplied regarding data availability: The code is available as a Supplemental File.
